# Considerations in hiPSC-derived cartilage for articular cartilage repair

**DOI:** 10.1186/s41232-018-0075-8

**Published:** 2018-10-04

**Authors:** Akihiro Yamashita, Yoshihiro Tamamura, Miho Morioka, Peter Karagiannis, Nobuyuki Shima, Noriyuki Tsumaki

**Affiliations:** 1 Cell Induction and Regulation Field, Department of Clinical Application, Center for iPS Cell Research and Application, Kyoto University, Kyoto, Japan; 2 International Public Communications Office, Center for iPS Cell Research and Application, Kyoto University, Kyoto, Japan

**Keywords:** Chondrocytes, Articular cartilage, Regeneration, Induced pluripotent stem cells, Transplantation

## Abstract

**Background:**

A lack of cell or tissue sources hampers regenerative medicine for articular cartilage damage.

**Main text:**

We review and discuss the possible use of pluripotent stem cells as a new source for future clinical use. Human induced pluripotent stem cells (hiPSCs) have several advantages over human embryonic stem cells (hESCs). Methods for the generation of chondrocytes and cartilage from hiPSCs have been developed. To reduce the cost of this regenerative medicine, allogeneic transplantation is preferable. hiPSC-derived cartilage shows low immunogenicity like native cartilage, because the cartilage is avascular and chondrocytes are segregated by the extracellular matrix. In addition, we consider our experience with the aberrant deposition of lipofuscin or melanin on cartilage during the chondrogenic differentiation of hiPSCs.

**Short conclusion:**

Cartilage generated from allogeneic hiPSC-derived cartilage can be used to repair articular cartilage damage.

## Background

Articular cartilage covers the ends of bones and composes joints, providing lubrication between opposing bones during joint motion. Cartilage is avascular and consists of chondrocytes embedded in abundant extracellular matrix (ECM), in which collagen fibrils form three-dimensional (3D) networks that provide scaffolding for proteoglycan. One function of cartilage ECM is to confer mechanical properties to cartilage tissue in order to sustain smooth joint motion. Chondrocytes and cartilage ECM have a mutually dependent relationship: Chondrocytes produce and maintain ECM, and ECM is necessary for the chondrocytes to sustain their chondrocytic property including the production of cartilage ECM. This mutual relationship is indispensable for the homeostasis of cartilage. Cartilage, when damaged through trauma, has only limited capacity for repair, probably because the damage causes a loss of cartilage ECM, disrupting the chondrocytic environment. The continued use of joints with damaged cartilage and poor repair capacity gradually expands the damaged area on the joint surface, resulting in debilitating conditions such as osteoarthritis.

### Current regenerative treatments for articular cartilage damage

Microfracture is the preferred treatment when the size of the articular cartilage defect is relatively small (less than 2–4 cm^2^). In this treatment, the damaged area of the cartilage is removed to create a defect, and small holes are made through the subchondral bone, which allows bone marrow cells to fill the defect. However, the resulting repair tissue made by the bone marrow cells is fibrous, which is not as functional as articular cartilage. Mosaicplasty is another preferred treatment for small defects. Here, multiple autologous cylindrical osteochondral grafts are harvested from the periphery of the articular surface and implanted into the damaged area. Mosaicplasty has the advantage of transplanting viable hyaline-like cartilage. Nevertheless, this technique is restricted by the availability of harvestable autologous graft and by the donor-site morbidity [1]. For larger articular cartilage defects, autologous chondrocyte transplantation (ACI) is preferred. Here, a small piece of cartilage is harvested from the periphery of the articular surface and subjected to treatment for the isolation of its chondrocytes. These chondrocytes are then expanded in monolayer culture and transplanted into the damaged area [2]. Although ACI provides good clinical results, it has limitations. The isolation of chondrocytes from cartilage ECM in culture causes a loss of the chondrocytic property and results in the conversion of the chondrocytes to fibroblastic cells [3–5]. Thus, the repaired tissue includes fibrous tissue, which has inferior joint function compared with articular cartilage [6]. Further, the transplanted cells constitute only a limited portion of the repaired tissue, while the remainder is composed of host cells. In fact, the benefit of the transplanted cells may partly come from the secretion of factors that stimulate the host cells, i.e., trophic effects. In addition to the physiology of the repair, another demerit of ACI is that patients are burdened with sacrificing donor sites and two-stage surgery.

Mesenchymal stem cells (MSCs) are alternative cell sources for cartilage repair. MSCs can be obtained from the bone marrow, adipose tissue, and synovium. Although MSCs have an ability to be differentiated toward chondrocytes, this ability tends to be lost after expansion [7]. Evidence that transplanted MSC-derived chondrocytes constitute repaired cartilage in vivo is scant, and, like ACI, the effects of the transplanted MSCs are considered trophic, in which secreted factors from the MSCs stimulate host cells to repair the tissue [8–10].

Because the repair mechanism of ACI and MSC transplantation involves trophic factors that act on host cells, microfracture is often employed with these methods to provide host cells from the bone marrow. However, whatever the stimulation, there is a limitation in the quality of the repair tissue so long the treatment depends on host cells for the creation of repair tissue.

The transplantation of allogeneic cartilage could resolve the scarcity of cells and the poor chondrocytic property of the transplants. Allogeneic cartilage transplantation is distinct from ACI or MSC transplantation in that the transplants are not mere cells, but actual cartilage tissue that can constitute most of the repair. Cartilage is considered immunoprivileged tissue [11, 12] because it lacks vasculature and because chondrocytes are embedded in the ECM, protecting the cells from immunological reactions. Indeed, allogeneic cartilage harvested from juveniles [12–16] have been transplanted successfully to treat defects. However, the lack of donors, the heterogeneous quality of the cartilage, and the risk of disease transmission are all limitations associated with allogeneic sources.

### Chondrocytes and cartilage generated from pluripotent stem cells as a source for regenerative medicine

The scarcity of allogeneic sources could potentially be resolved with human pluripotent stem cells (hPSCs) such as human embryonic stem cells (hESCs) [17] and human induced pluripotent stem cells (hiPSCs) [18]. ESCs and iPSCs share characteristic properties, such as pluripotency and self-renewal, and can be maintained in identical culture conditions. However, they differ in their preparation: ESCs are acquired from inner cell mass of embryos, while iPSCs are somatic cells, such as skin cells or blood cells, that have been reprogrammed to the pluripotent state by the introduction of specific factors [19]. Methods for the differentiation of both cells toward chondrocytes have been developed and are interchangeable. Because hESCs and hiPSCs can be expanded almost infinitely due to their self-renewal capacity, a large number of chondrocytes can be prepared. In fact, it is now possible to generate enough chondrocytes of good quality for regenerative medicine at the experimental level, although several issues must be resolved before translating these experimental findings to the clinic.

### Autologous vs. allogeneic transplantation

One advantage of hiPSCs over hESCs is that their creation does not involve the destruction of an embryo, thus avoiding certain ethical controversies. Another merit is that hiPSCs can be made from the patient’s own cells, permitting the possibility of autologous transplants [20]. However, the preparation of patient-iPSCs and subsequent differentiation under good manufacturing practice (GMP) guidelines is costly. To reduce the cost and to provide treatment to a large population, a bank of allogeneic clinical GMP grade hiPSC lines is being established [21, 22]. To reduce the risk of immune rejection during the transplantation of tissues generated from allogeneic hiPSCs, this iPSC library is prepared from donors homozygous for major HLA types. It is much easier to prepare homozygous HLA hiPSCs than hESCs, because it is easier to find individuals who bear homozygous HLA types and are willing to donate their somatic cells for iPSC generation compared with embryos for ESC generation. It is estimated that a bank of 1, 50, and 140 cell lines homozygous for major HLA types from Japan would respectively match 17, 73, and 90% of the population [22].

As explained above, cartilage is considered to have low immunogenicity, and the transplantation of allogeneic cartilage has been performed in a large number of patients without matching for HLA types and without the administration of immunosuppressive drugs. The transplantation of allogeneic, particulated juvenile articular cartilage has given good clinical results [14], although the long-term clinical outcome remains to be investigated. Cartilage can be generated from hiPSCs by making hiPSC-derived chondrocytes that produce and deposit ECM around themselves in 3D culture [23, 24]. The avascular structure of cartilage and ECM produced from the chondrocytes prevent a recipient’s immune cells from contacting the chondrocytes in the transplanted hiPSC-derived cartilage. Mixed lymphocyte reaction assays have shown that hiPSC-derived cartilage has the low immunogenicity of human cartilage [25]. Moreover, hiPSC-derived chondrocytes are more similar to juvenile chondrocytes than to adult chondrocytes, and it has been reported that cartilage from juveniles have more anabolic activity and are less antigenic than those from adults [12, 16, 26]. These findings imply that cartilage prepared from a single allogeneic hiPSC or hESC clone could be used for all patients, which would standardize the quality and lower the cost of this regenerative medicine.

### Generation of cartilage from hiPSCs

The basic principle in currently available protocols for the chondrogenic differentiation of PSCs is to direct cell fate to chondrocytic lineage and to eliminate non-chondrocytic cells [20, 27–29]. To realize this scheme, the composition of the culture medium and supplements, including growth factors such as TGF-β, BMP, WNT, and FGF; the cell density; and the coating of the dishes on which the cells are grown must be considered.

**Fig. 1:**
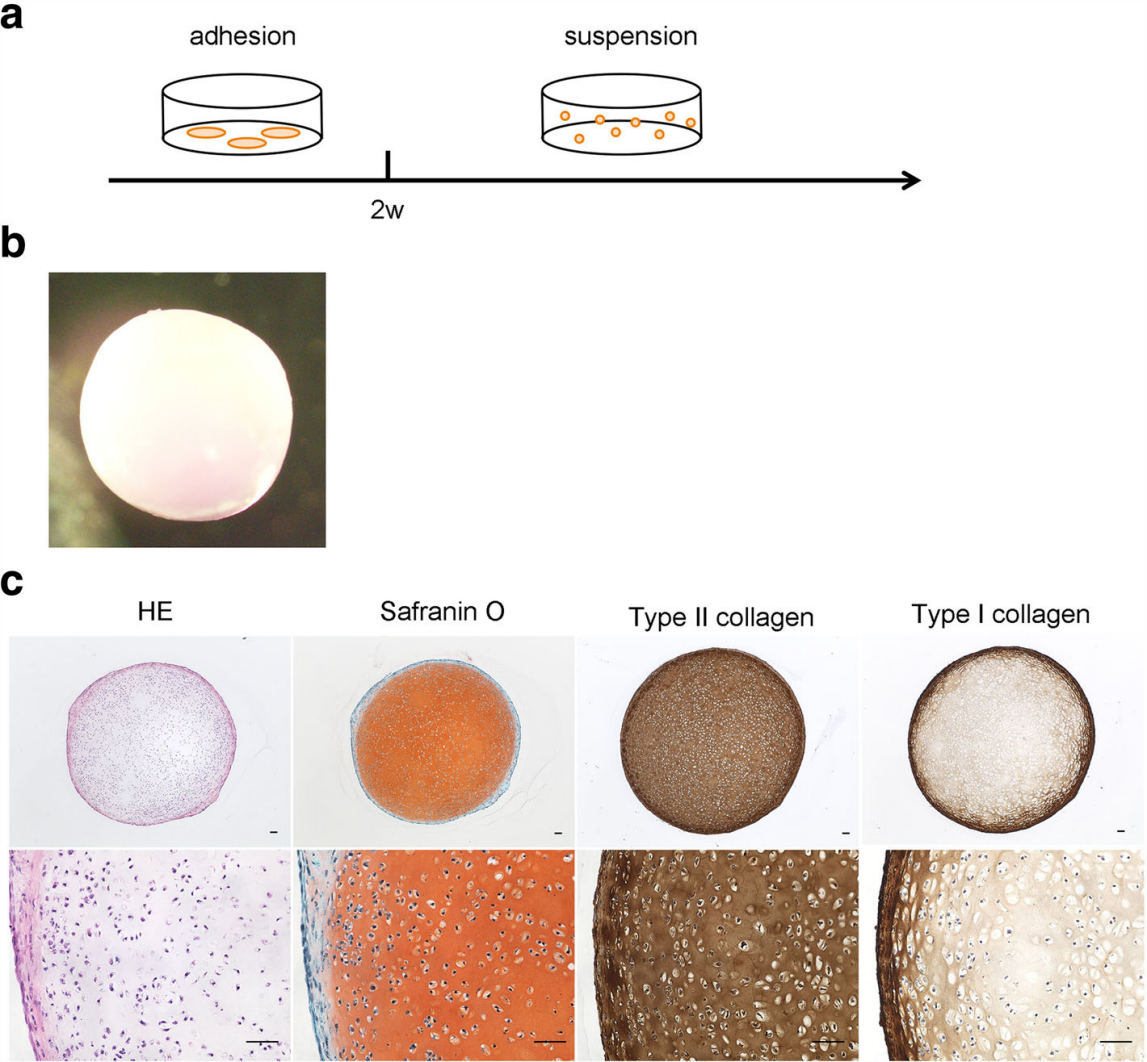
Generation of cartilaginous particles from hiPSCs. **a** Scheme of the chondrogenic differentiation of hiPSCs. **b** Image of a hiPSC-derived cartilaginous particle at 12 weeks. **c** Histological analysis of the hiPSC-derived cartilaginous particle at 12 weeks. Semiserial sections were stained with hematoxylin-eosin and safranin O-fast green-iron hematoxylin and immunostained with anti-type II collagen antibodies and anti-type I collagen antibodies. Bars, 50 μm.

We previously developed a method to generate cartilage from hiPSCs [23, 24]. hiPSCs in adhesion culture were initially differentiated into mesendodermal cells in the presence of WNT and Activin. Then, the cells were differentiated into chondrocytes in chondrogenic medium containing TGF-β, BMP-2, and GDF-5. The resulting chondrocytes were subsequently transferred into 3D suspension culture in which the chondrocytes secreted and accumulated cartilage ECM around themselves to create cartilaginous tissues that look like white particles of 2–3 mm diameter (Fig. 1a, b). Histological analysis of each particle showed that the particles consisted of central cartilaginous tissue and were surrounded by membranous tissue (Fig. 1c). This structure may correspond to cartilage surrounded by perichondrium. Although the observation periods are limited, transplantation of the hiPSC-derived cartilage into articular cartilage defects in immunodeficient rats and immunosuppressed mini-pigs showed that the transplanted cartilage survived and had potential for integration into native cartilage [24]. These results suggest that hiPSC-derived cartilage can be used to repair articular cartilage damage.

### Aberrant deposition of lipofuscin or melanin during chondrogenic differentiation of hiPSCs

**Fig. 2:**
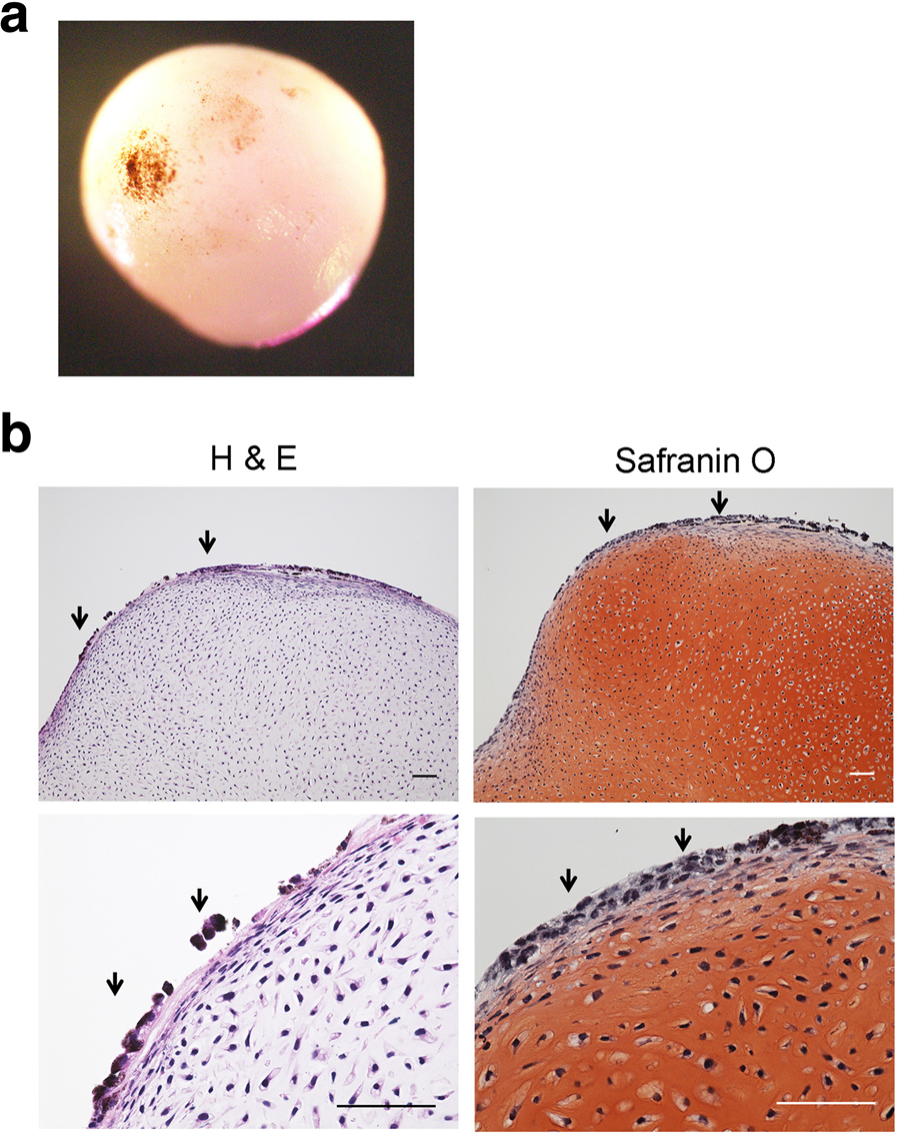
Aberrant emergence of dark dots on the surface of hiPSC-derived cartilaginous particles. **a** Image of a hiPSC-derived cartilaginous particle with dark dots at 12 weeks. **b** Histological analysis of the hiPSC-derived cartilaginous particle in **a**. Semiserial sections were stained with hematoxylin-eosin and safranin O-fast green-iron hematoxylin. Arrows indicate dark dots. Bars, 50 μm.

**Fig. 3:**
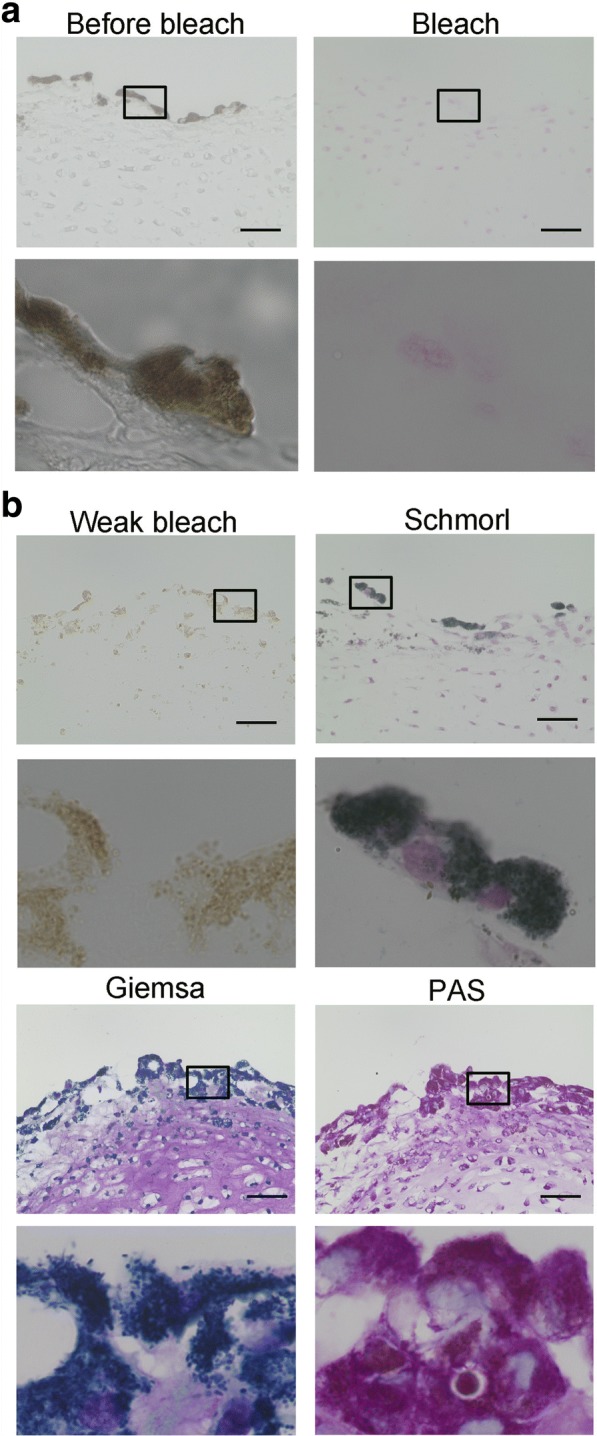
Analysis of dark dots on hiPSC-derived cartilaginous particles. Histological analysis of a hiPSC-derived cartilaginous particle with dark dots at 12 weeks. **a** Sections were bleached and counterstained with kernechtrot. **b** Sections were initially bleached weakly and subjected to PAS staining, Giemsa staining, and Schmorl’s reaction. Bars, 50 μm. Boxed regions are magnified in the images below.

Protocols for chondrogenic differentiation should be robust and produce cartilage of high quality. However, it is known in the field that culture does not always go as expected. As an example, we have observed unexplained dark dots on the surface of some hiPSC-derived cartilaginous particles on rare occasion (Fig. 2a). This phenomenon was observed in several independent hiPSC lines. The presence of malaria pathogens and formalin is known to cause artificial dark pigments (Table 1), but culture conditions are free of these substances. Thus, to explain these dots, we investigated natural pigmentations (Table 1) [30]. Histological analysis revealed that the dark dots resided in the cytoplasm of cells located on the surface of the particles (Fig. 2b). The dark dots lost color after bleaching by treatment with potassium permanganate, suggesting they are not hematogenous pigments, which are resistant to bleaching [31] (Fig. 3a). Among non-hematogenous pigments, melanin, chromaffin, and lipofuscin look dark and are sensitive to bleach [32, 33]. These three pigments can be discriminated by reactions to PAS staining, Schmorl’s reaction, and Giemsa staining (Table 2) [32–35]. Further investigation found that the dark dots were positively stained with Schmorl [34]. Giemsa staining indicated dark blue [35], but we could not rule out that it indicated dark green (Fig. 3b). The area stained by PAS overlapped the dark dots, although not completely. Based on these findings, we considered the dark dots on the surface of hiPSC-derived cartilage to be either lipofuscin or possibly melanin. Either case could compromise the use of these particles for regenerative medicine.

**Table 1:** Pigments

Source	Pigment type	Color
Hematogenous	Hemoglobin	Red to brown
	Hemosiderin	Yellow to brown
	Bile	Yellow to brown
	Porphyrin	Dark brown
Non-hematogenous	Melanin	Brown to black
	Chromaffin	Dark brown
	Lipofuscin	Yellow to brown
Artifact	Malaria	Dark brown
	Formalin	Dark brown

**Table 2:** Pigments and staining methods

	Non-hematogenous pigment			Hematogenous pigment
Staining	Lipofuscin	Melanin	Chromaffin	
Bleach	Sensitive	Sensitive	Weakly sensitive	Resistant
PAS	Positive	Negative	Negative	Negative
Giemsa	Ortho-chromasia (dark blue)	Meta-chromasia (dark green)	Meta-chromasia (red purple)	
Schmorl’s reaction	Positive	Positive	Weakly positive	
Berlin blue	Negative	Negative	Negative	Positive (hemosiderin)

Lipofuscin is a cross-linked aggregate that consists of oxidized proteins and lipids and is resistant to cellular proteolytic systems [36, 37]. Lipofuscin is insoluble and is not exocytosed by cells such that it resides in the cytosol. Lipofuscin is found in postmitotic cells in various tissues and organs and has two sources. One is damaged mitochondria caused by a malfunction of the mitochondrial repair system due to aging and/or excess amounts of reactive oxygen species (ROS). The other is damaged proteins, such as oxidized proteins and unfolded proteins that cross-link in the cytosol. Normally, these damaged particles are taken up by lysosomes and degraded. A failure in this degradation leads to the damaged mitochondria and proteins within the lysosomes cross-linking and binding more particles, such as lipids, to produce lipofuscin. Lipofuscin enters the cytosol if the lysosomes rupture. There, lipofuscin may disturb cell function and consume both lysosomal enzyme capacity and lysosomal space, further reducing lysosomal degradation capacity. Furthermore, it may have a chemically reactive surface that can disturb cellular metabolism. Lipofuscin has been reported to accumulate in natural cartilage [38]. Although the effects of lipofuscin on cartilage metabolism are unknown, culture conditions that produce no lipofuscin should be used for repairing cartilage damage.

The mechanism by which dark dots aberrantly form during the chondrogenic differentiation of hiPSCs remains to be elucidated. We found the dark dots reproducibly appeared when we increased cell density during the differentiation of hiPSCs toward chondrocytes. Consistently, dark dots did not appear when the differentiation protocol was done without cell overgrowth. It has been reported that lipofuscin forms in the chondrogenic micromass culture of chick limb bud mesenchymal cells when they are treated with agents that increase the generation of OH(.) radicals [39]. Whether the generation of OH(.) radicals contributes to the dark dots seen with cell overgrowth during the chondrogenic differentiation of hiPSCs remains to be determined.

On the other hand, if the dark dots are due to the presence of melanin, then it is likely that a differentiated subpopulation took the melanocyte fate. It is unknown whether here too cell densities could have an effect. The contamination of melanocytes in the hiPSC-derived cartilage would inevitably compromise the repair process after transplantation because this subpopulation does not have chondrogenic function. Whichever causes the dark dots (lipofuscin or melanin), cartilage particles generated from iPSCs should avoid their presence when considering the repair of articular cartilage damage.

## Conclusions

Cartilage is a tissue that consists of chondrocytes and ECM, which are mutually dependent. The healing mechanisms by cell transplantation such as ACI or MSC transplantation into articular cartilage defects may depend on trophic effects, whereas the transplantation of cartilage tissue can produce repair tissue of good quality. hiPSCs promise a new generation of cartilage therapy. Cartilage tissue can be produced by the differentiation of hiPSCs into chondrocytes followed by transferring the cells into 3D suspension culture in which the chondrocytes secrete and accumulate surrounding cartilage ECM. hiPSC-derived cartilage has low immunogenicity like natural cartilage and can be transplanted in an allogeneic manner for the treatment of articular cartilage damage. The differentiation of iPSCs to cartilage should be tightly controlled to avoid the presence of non-cartilaginous elements such as lipofuscin or melanin.

## Methods

### Chondrogenic differentiation of hiPSCs

Cartilaginous particles were generated from hiPSCs as described previously [24] with modification. hiPSCs-derived cartilaginous particles generated 12 weeks after the start of the differentiation were used.

### Histological analysis

hiPSC-derived cartilaginous particles were fixed with 4% paraformaldehyde, processed, and embedded in paraffin. Semiserial sections were stained with hematoxylin-eosin and safranin O-fast green-iron hematoxylin and immunostained with anti-type II collagen antibody and goat anti-type I collagen antibody, as described previously [24].

Semiserial sections were bleached with 0.25% potassium permanganate (Nacalai) for 1 h, then with 2% oxalic acid for 2 min, and finally counterstained with kernechtrot (Muto pure chemicals).

For Schmorl’s reaction, PAS staining, and Giemsa staining, semiserial sections were initially weakly bleached with 0.25% potassium permanganate (Nacalai) for 3 min and 2% oxalic acid for 2 min. The sections were subjected to Schmorl’s reaction by being stained with ferric chloride (Wako) and counterstained with kernechtrot. The sections were subjected to PAS staining by being treated with Schiff’s reagent (Wako) for 10 min and counterstained with hematoxylin. Finally, the sections were subjected to Giemsa staining by being stained with Giemsa stain solution composed of Giemsa (Merck), methanol, and sodium carbonate (Nacalai) for 3 h.
